# Structural equation modeling in medical research: a primer

**DOI:** 10.1186/1756-0500-3-267

**Published:** 2010-10-22

**Authors:** Tanya N Beran, Claudio Violato

**Affiliations:** 1 Medical Education and Research Unit Faculty of Medicine University of Calgary 3330 Hospital Dr. N.W. Calgary, AB T2N 4N1, Canada

## Abstract

**Background:**

Structural equation modeling (SEM) is a set of statistical techniques used to measure and analyze the relationships of observed and latent variables. Similar but more powerful than regression analyses, it examines linear causal relationships among variables, while simultaneously accounting for measurement error. The purpose of the present paper is to explicate SEM to medical and health sciences researchers and exemplify their application.

**Findings:**

To facilitate its use we provide a series of steps for applying SEM to research problems. We then present three examples of how SEM has been utilized in medical and health sciences research.

**Conclusion:**

When many considerations are given to research planning, SEM can provide a new perspective on analyzing data and potential for advancing research in medical and health sciences.

## Text

Structural equation modeling (SEM) is a powerful multivariate analysis technique that is widely used in the social sciences [1]. Its applications range from analysis of simple relationships between variables to complex analyses of measurement equivalence for first and higher-order constructs [2]. It provides a flexible framework for developing and analyzing complex relationships among multiple variables that allow researchers to test the validity of theory using empirical models. Perhaps its greatest advantage is the ability to manage measurement error, which is one of the greatest limitations of most studies. Although its application has been seen in many disciplines, it has yet to be extensively used in medical research and epidemiology.

In a recent paper, we provided a "how to" for medical education researchers [3]. Specific principles and examples for the field of medical education were utilized. The purpose of the present paper, however, is to introduce structural equation modeling through explanation and demonstration of its methods in an attempt to disseminate it more widely in medical and health sciences research.

The use of SEM has now become widespread across research domains. In psychology, for example, the citation frequency of SEM has steadily increased from 164 in 1994 to 343 in 2000 and then to 742 in the last year (based on the citation frequency of SEM and M[ANOVA] of PsychINFO database 1970-2010) [4,5]. This suggests that researchers recognize its application to a variety of research questions, types of data, and methods of study. An increase in use of sophisticated tools of analysis reflects the increase in complexity of empirical models and theoretical developments seen in the published research over the years.

In a recent (2009) commentary in the *International Journal of Epidemiology*, Tu expressed concern about the scarcity of SEM models in epidemiological research and urged epidemiologists to use SEM models more frequently [6]. With its strength as a statistical tool to analyze complex relationships among variables, and even posit and test causal relationships with non-experimental data, it allows researchers to explain the development of phenomena such as disease and health behaviors. The purpose of the present paper is to consider the potential advances that SEM can make in medical and health sciences research and provide a five step approach to implementing SEM research in epidemiology and medical research. First a description of SEM is provided, followed by applications to research. A broad categorization of statistical methods is termed 'latent variable models', which include factor analysis, item response theory, latent class models, and structural equation models [7]. The focus of the present paper in on structural equation models and the latent variable models that are included in SEM.

## 1. Description of SEM

Although SEM was developed in the early 1900s as a result of Spearman's (1904) development of factor analysis and Wright's (1918, 1921) invention of path analysis, the first basic introductory textbook on SEM was not published until 1984 [8-11]. With the advances in computer programming such as EQS (EQuationS implementing Structural Equation Modeling), and LISREL (Linear Structural Relationships) researchers began utilizing SEM techniques in their research [12,13]. Indeed, it has become "the preeminent multivariate technique" [4] and is now accessible on-line at no cost (e.g., http://openmx.psyc.virginia.edu/).

There are several integrated analytic techniques within SEM. These include between-group and within-group variance comparisons, which are typically associated with ANOVA. It also includes path analysis (regression analysis) whereby equations representing the effect of one or more variables on others can be solved to estimate their relationships.^a ^Path analysis, thus, represents the hypothesized causal relationships among variables to be tested. Factor analysis is another special case of SEM whereby unobserved variables (factors or latent variables) are calculated from measured variables. These analyses can usually be performed using data in the form of means or correlations and covariances (i.e., unstandardized correlations). These data, moreover, may be obtained from experimental, nonexperimental and observational studies. All of these techniques can be incorporated into the following example.

Several symptoms of a disease are measured and used in a factor model that represents these symptoms. The relationship between the factor(s) and behavioral and/or environmental characteristics are determined through path analysis. The impact of different types of medication on the factor(s) is then compared across the measured behavioral and environmental conditions.

To conduct the above analyses, both a structural (i.e., path) and a measurement model are designed by the researcher. The structural model refers to the relationships among latent variables, and allows the researcher to determine their degree of correlation (calculated as path coefficients). That is, path coefficients were defined by Wright (1920, p. 329) as measuring the importance of a given path of influence from cause to effect [14]. Each structural equation coefficient is computed while all other variances are taken into account. Thus, coefficients are calculated simultaneously for all endogenous variables rather than sequentially as in regular multiple regression models.

**Figure 1:**
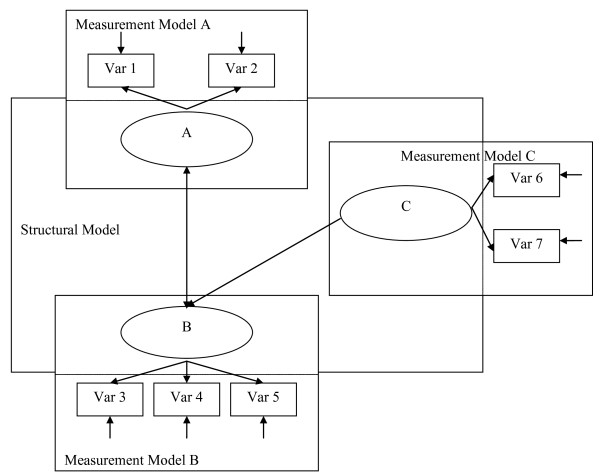
**A structural equation model - from Nachtigall C, Kroehne U, Funke F, Steyer R**. **Why should we use SEM? Pros and cons of structural equation modeling**. *Meth Psychol Res Online *2003, **8**:1-22.

To determine the magnitude of these coefficients, the researcher specifies the structure of the model. This is depicted in Figure 1. As shown, the researcher may expect that there is a correlation between variables A and B, as shown by the double headed arrow. There may be no expected relationship between variables A and C, so no arrow is drawn. Finally, the researcher may hypothesize that there is a unidirectional relationship of variable C to B, as indicated by an arrow pointing from C to B. The relationships among variables A, B, and C represent the structural model. Researchers detail these relationships by writing a series of equations, hence the term 'structural equation' (referring to the relationships between the variables). The combination of these equations specifies the pattern of relationships [12].

The second component to be specified is the measurement model. As represented in Figure 1, it consists of the measured variables (e.g., variables 1-7), which are typically used in research, as well as latent variables. Latent variables are factors like those derived from factor analysis, which consist of at least two inter-related measured variables. They are called latent because they are not directly measured, but rather are represented by the overlapping variance of measured variables. They are said to better represent the research constructs than are measured variables because they contain less measurement error. As indicated in Figure 1, for example, measurement model A depicts a latent variable A, which is the construct underlying measured variables 1 and 2. To further explicate the process of developing and analyzing a model, the following steps are outlined next.

### Step 1: Identify the Research Problem

The researcher develops hypotheses about the relationships among variables that are based on theory, previous empirical findings or both [15]. These relationships may be direct or indirect whereby intervening variables may mediate the effect of one variable on another. The researcher must also determine if the relationships are unidirectional or bidirectional, by using previous research and theoretical predictions as a guide. The researcher outlines the model by determining the number and relationships of measured and latent variables. Care must be taken in using variables that provide a valid and reliable indicator of the constructs under study. The use of latent variables is not a substitute for poorly measured variables. A path diagram depicting the structural and measurement models will guide the researcher when identifying the model, as described next.

### Step 2: Identify the Model

Identifying the model is a crucial step in model development as decisions at this stage will determine whether the model can be feasibly evaluated. For each parameter in the model to be estimated, there must be at least as many values (i.e., variance and covariance values) as model parameters (e.g., path coefficients, measurement error).^b ^A model that has fewer of these values than parameters is referred to as underidentified and impossible to solve mathematically. This problem also occurs when variables are highly intercorrelated (multicollinearity)^c^, the scales of the variables are not fixed (the path from a latent variable to one of the measured variables must be set as a constant), or there is no unique solution to the equations because the underidentification results in more parameters to be estimated than information provided by the measured variables. In underidentified models there are an infinite number of solutions and therefore no unique one. These problems may be remedied with the addition of independent variables, which requires that the model be conceptualized before data are collected. There are many further issues to consider when managing parameters that cannot be addressed in this primer. For further details on model identification, readers are encouraged to see Kline [16].

### Step 3: Estimate the Model

There are many estimation procedures available to test models, with three primary ones discussed here. ML is set as the default estimator in most SEM software. It is an iterative process that estimates the extent to which the model predicts the values of the sample covariance matrix, with values closer to zero indicating better fit. The name maximum likelihood is based on its calculation. The estimate maximizes the likelihood that the data were drawn from its population. The estimates require large sample sizes, but do not usually depend on the measurement units of the measured variables. It is also robust to non-normal data distributions [17].

Another widely used estimate is least squares (LS), which minimizes the sum of the squares of the residuals in the model. LS is similar to ML as it also examines patterns of relationships, but does so by determining the optimum solution by minimizing the sum of the squared deviation scores between the hypothesized and observed model. It often performs better with smaller sample sizes and provides more accurate estimates of the model when assumptions of distribution, independence, and asymptotic sample sizes are violated [18].

The third, asymptotically distribution free (ADF) estimation procedures (also known as Weighted Least Squares) are less often used but may be appropriate if the data are skewed or peaked. ML, however, tends to be more reliable than ADF. This method also requires sample sizes of 200 to 500 to obtain reliable estimates for simple models and may under-estimate model parameters [16,19]. For further details see Hu et al. [19] and Muthén and Kaplan [20].

### Step 4: Determine the Model's Goodness of Fit

These estimation procedures determine how well the model fits the data. Fitting the latent variable path model involves minimizing the difference between the sample covariances and the covariances predicted by the model. The population model is formally represented as:

(1) Σ=Σ(θ)

Where **Σ **is the population covariance matrix of observed variables, **θ **is a vector that contains the model parameters, and **Σ (θ) **is the covariance matrix written as a function of **θ**. This simple equation allows the implementation of a general mathematical and statistical approach to the analysis of linear structural equation system through the estimation of parameters and the fitting of models. Estimation can be classified by type of distribution (multinormal, elliptical, arbitrary) assumed of the data and weight matrix used during the computations. The function to be minimized is given by:

(2) Q=[s−σ(θ)]'W[s−σ(θ)]

where s is the vector of data to be modeled - the variances and covariances of the observed variables - and σ is a model for the data. The model vector σ is a function of more basic parameters θ that are to be estimated so as to minimize Q. **W **is the weight matrix that can be specified in several ways to yield a number of different estimators that depend on the distribution assumed.

Essentially the researcher attempts to represent the population covariance matrix in the sample variables. Then, an estimation procedure is selected, which runs through an iterative process until the best solution is found.

Another source of information in the output is the fit indices. There are many indices available, with most ranging from 0 to 1 with a high value indicating a great degree of variance in the data accounted for by the model [21]. The Comparative Fit Index (CFI) is most commonly used and compares the existing model with a null model. A good fit is also represented by low residual values (e.g., .00), which represents the amount of variance not accounted for by the model. These are calculated as indices such as the Root Mean Square Error of Approximation (RMSEA), which is the square root of mean differences between the estimate and the true value. Another goodness-of-fit statistic commonly reported is χ^2^, which assesses the likelihood that the differences between the population covariance matrix and model implied covariance matrix are zero. This statistic, however, varies as a function of sample size, cannot be directly interpreted (because there is no upper bound), and is almost always significant. It is useful, however, when directly comparing models on the same sample. Dahly, Adair, and Bollen [22], for example, tested various fit indices for different models depicting the relationship between maternal height and arm fat area with fetal growth. When adding and removing variables, as well as specifying varying relationships between variables, each corresponding fit index was calculated. This allowed the researchers to determine factors in the fetal environment that are most significantly related to systolic blood pressure of young adults. In summary, when evaluating fit statistics, CFI values ≥ .90 and RMSEA < .05 are considered adequate [23].

A comparison of indices was conducted by Hu and Bentler [18] on data that violated assumptions of normal distribution, independence of observations, and symmetry. Their results indicate that TLI, BL89, RNI, CFI, Mc, Gamma Hat, and RMSEA are able to identify good models. Many of these are provided by standard SEM software packages (e.g., EQS, LISREL, Mplus, AMOS).^d^

To determine the model's goodness-of-fit, sample size is an important consideration. It must be large enough to obtain stable estimates of the parameters. Many recommendations have been published, suggesting that there is no precise decision rule. Monte Carlo studies provide guidance that sample sizes of 10 for a one-factor, five-observed variable model, and 30 for a two-factor, five-observed variable model provide robust results [24]. More general guidelines are used in current research with the suggestion that at least 100 but preferably 200 cases are needed to obtain stable results [16]. Using a large sample reduces the likelihood of random variation that can occur in small samples [25], but may be difficult to obtain in practice.

### Step 5: Re-specify the Model if Necessary

To obtain improved fit results, the above sequence of steps is repeated until the most succinct model is derived (i.e., principle of parsimony). A recommended procedure to improve the model estimations is through examination of the size of the standardized residual values between variables. Large residuals may suggest inadequate model fit. This can be addressed by the addition of a path link, or inclusion of mediating or moderating variables (if theoretically supported). Once the model is re-calculated, its fit may show improvement and residual may be reduced. These results then need to be confirmed on an alternate sample, and through further studies. This replication strengthens confidence in the inferences, and provides implications for theoretical development and practical application.

### Further Considerations

Before executing SEM procedures, there are many additional topics to consider. As for any research study, careful planning of design, sampling, and measures is needed to develop valid models. SEM can be used in either cross-sectional or longitudinal studies, whereby the former are identified by links among variables measured at the same point in time, and the latter are specified by the links among variables measured at different points in time. These models often include autoregressive effects where a variable measured at two time points is correlated with itself. This corrects for an over-estimation of the relationship among exogenous (independent) and endogenous (dependent) variables [26]. While this is a distinct advantage of SEM, it is often disregarded.

Types of measures must also be considered [27]. Calculations of variances, covariances and product-moment correlations all assume that values are measured on an interval scale. Measures that include, for example, rating scales without equal distances between data points, are not necessarily considered appropriate [28]. Researchers must be prudent in selecting the appropriate procedures for particular levels of measurement including, for example, dichotomous and polytomous data [29]. Indeed, another advantage of SEM is the ability to manage continuous and binary data simultaneously.

SEM can be employed for both exploratory and confirmatory models. An exploratory approach is more traditional in that a detailed model specifying the relationships among variables is not made a priori. All latent variables are assumed, therefore, to influence all observed variables so that the number of latent variables are not pre-determined, and measurement errors are not allowed to correlate [29]. Although both exploratory and confirmatory factor analyses are a subset of SEM involving the measurement model only, the latter is more frequently used to test hypothetical constructs. The following section presents three examples of application of SEM in medical and health sciences research.

## 2. Examples of SEM

SEM has been applied in psychiatry to understanding patients' experiences of schizophrenia. Loberg and colleagues examined the role of positive symptoms and duration of schizophrenia on dichotic listening of patients [30]. Dichotic listening tasks are used as a means of assessing functioning within the left temporal lobe language areas. Previous research suggested increased impairment in left temporal lobe language processing among patients with a high number of positive symptoms (e.g., hallucinations and delusions) of schizophrenia.

Loberg, Jorgensen, Green, Rund et al [30] attempted to replicate these results as well as determine whether duration of illness further decreases language functioning. A total of 129 patients from clinics in Norway and California diagnosed with schizophrenia were included.^e ^All patients were taking haloperidol (an antipsychotic) or an equivalent.

**Figure 2:**
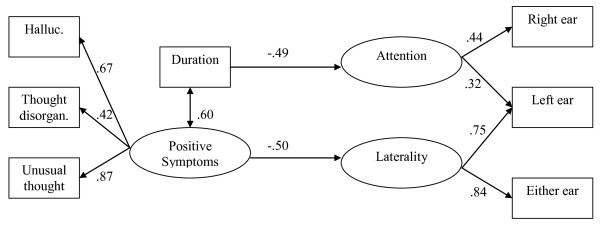
**Model of positive symptoms, duration of schizophrenia, and dichotic listening**.

The Extended Brief Psychiatric Rating Scale and Positive and Negative Syndrome scale were completed by blind observers to measure symptoms of schizophrenia, and the duration of the disease was calculated based on initial onset of symptoms. Dichotic listening was measured by patients' responses to consonant and vowel blends spoken through headphones. In one condition patients were told which ear to listen with (attention) and in another they were not (laterality). The theoretical model tested is shown in Figure 2.

Analysis of this model using SEM indicated it fit the data well. The CFI was 0.986 based on 11 degrees of freedom. Close inspection of the model (Figure 2) shows that all the path coefficients to the predicted latent variables are moderate to high (range from .32 to .87). The RMSEA was .048. Positive symptoms were measured by hallucinations, disorganized thoughts, and unusual thought content. Dichotic listening was measured by accuracy of sounds identified in each ear according to the condition in which the patients heard the sounds.

In terms of the relationship between dichotic listening and schizophrenia, duration of schizophrenia and number of positive symptoms were related to accuracy of sound detection. That is, patients who have had schizophrenia for a longer duration and experience more positive symptoms, the poorer their identification of vowel-consonant blends. These results support findings from previous research suggesting impaired language processing and structural abnormalities in the left superior temporal gyrus for patients with schizophrenia.

The advantage of this research over other studies is that it examines three types of positive symptoms and duration of schizophrenia simultaneously, rather than separately, in relation to dichotic listening. In other words, the model also suggests that patients with many positive symptoms are likely to have difficulty identifying sounds accurately, especially if the duration of the illness is long. Greater confidence can be placed in these results than other regression models because more than one indicator of the constructs of interest was used in the model. Identifying basic underlying latent variables (positive symptoms and dichotic listening) is another advantage over interpreting simple correlations among measured variables.

Because this is a cross-sectional model, it is unknown whether the language processing deficit existed before, at the same time, or after the onset of schizophrenia. Direction of cause in the model is, thus, unknown. Given that time was an important variable in this model, we can explore the advantages of longitudinal modeling, or measuring variables at more than one point in time. That is, using the same measures of positive symptoms of schizophrenia and language processing taken at Time 1 and Time 2, path coefficients between the two latent variables at both points in time can be simultaneously examined to determine those that are significant. Previously a cross-lagged design would have been used whereby positive symptoms at Time 1 are correlated with language processing at Time 2. This correlation is then compared to the correlation between language processing at Time 1 and positive symptoms at Time 2. This comparison does not account for autoregression, does not include latent variables, and cannot be easily applied to multiple time points or multiple variables. An alternate method is multiple regression analysis whereby the positive symptoms of schizophrenia and language processing measured at Time 1 are used to predict language processing at Time 2. The magnitude of the regression weights would indicate the strength of the relationship between schizophrenia and language processing while controlling for initial language processing. Although this takes autoregression into account and includes multiple measured variables, latent variables cannot be used, and reciprocal patterns (impact of language processing on positive symptoms) cannot be examined.

A second example of SEM is of a model in population health that depicts the relationship between childhood victimization and school achievement. Beran and Lupart postulated that children who are targeted by acts of aggression from their peers may be at risk for poor achievement [31]. This argument is supported by Eccles' Expectancy-Value theory [32]. Accordingly, achievement involves the culture, socialization, and the environmental "fit" of schools for students. When children are exposed to positive experiences within this environment they are likely to gain academic and social competence [33]. Exposure to aggressive initiations from peers, however, may reduce a child's sense of competence for interpersonal interactions. Given that learning at school takes place in a social environment these harmful interactions may reduce learning behaviors such as volunteering answers and asking questions. Rather, children who are targeted may become discouraged and disengaged from peers and classroom learning [33].

**Figure 3:**
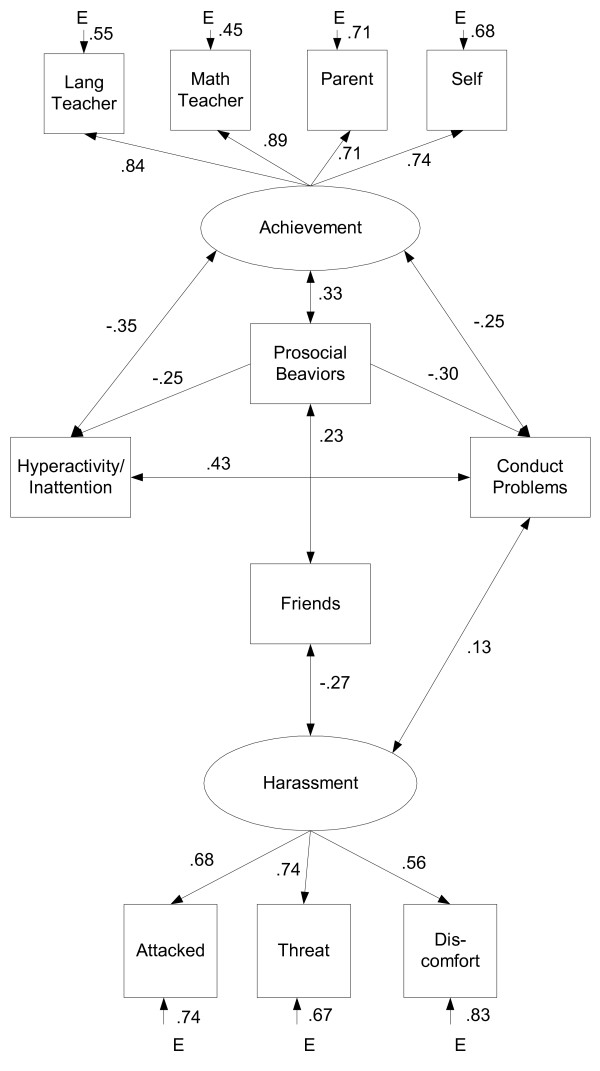
**Latent variable path model of harassment and achievement employing maximum likelihood estimation (*n *= 613)**.

In further developing their model, Beran and Lupart [31] included several correlates of achievement reported in previous research: impaired peer social skills (helping others), limited friendships (feeling disliked), and disruptive behaviors (aggression towards others, hyperactivity/inattention). All of these factors were simultaneously examined to determine the likelihood of targeted adolescents experiencing poor achievement. The theoretical SEM model is depicted in Figure 3.

Adolescents between 12-15 years of age (n = 4,111) were drawn from the Canadian National Longitudinal Survey of Children and Youth, which is a stratified random sample of 22,831 households in Canada [34]. As shown in Figure 3, harassment was related to disruptive behavior problems and peer interactions, which were related to achievement, χ^2^(32) = 300.00, p < .001, SRMR = .05; CFI = .91. Achievement was measured by four report sources including the language arts and math teachers, who reported on performance in those subjects, and the parent and child's report of overall achievement. Victimization was measured by adolescents' reports of frequency of attack and threats received from peers as well as degree of discomfort they feel among their peers. Victimization and achievement were used as latent variables in the model and were found to be mediated by disruptive behaviors and friendship experiences. This is shown by the arrows and coefficients whereby there is no arrow directly linking victimization with achievement. Rather, harassment was related to friendships and conduct problems, indicating that adolescents who were harassed reported having few or no friends (as shown by the negative sign) and exhibited conduct problems. These conduct problems were related to hyperactivity/inattention and prosocial behaviors such that adolescents with more rule breaking tendencies were likely to demonstrate hyperactive and inattentive behaviors as well as few prosocial, or helping, behaviors. These factors were also related to achievement. These combined results suggest that adolescents who are targeted by their peers are at risk of experiencing poor school achievement if they exhibit disruptive behavior problems and poor peer interactions.

A third example applies SEM within the field of clinical epidemiology by examining how health nutrition behaviors can serve to reduce risk of illness within a senior population. Specifically, Keller [35] examined behaviors that constitute risk of poor nutrition among seniors as part of a screening intervention. A measurement model of risk factors that constitute poor nutrition was developed a priori based on exploratory results from a previous study that identified four factors from 15 measured variables. A total of 1,218 Canadian seniors were interviewed or self-administered 15 questions about eating behaviors that matched those used previously. Variables such as type and frequency of food eaten created the latent factor food intake; appetite and weight change loaded on the factor adaptation; swallowing and chewing ability loaded on the factor physiologic; and cooking and shopping ability formed the variable functional. These factors were then loaded onto a higher level factor nutritional risk. The model fit the data well according to the CFI (> .90) and the RMSEA (< .05). Factor loadings varied between .15 and .66. It was, thus, concluded that these factors provide a comprehensive and valid indicator of nutritional risk for seniors. This framework was developed from previous research and presents confirmatory evidence for the nutrition behaviors used in the model of nutrition risk.

## 3. Strengths and Weaknesses of SEM

### Strengths

SEM is a set of statistical methods that allows researchers to test hypotheses based on multiple constructs that may be indirectly or directly related for both linear and nonlinear models [36]. It is distinguished from other types of analyses in its ability to examine many relationships while simultaneously partialing out measurement error. It can also examine correlated measurement error to determine to what degree unknown factors influence shared error among variables - which may affect the estimated parameters of the model [37]. It also handles missing data well by fitting raw data instead of summary statistics. SEM, in addition, can be used to analyze dependent observations (e.g., twin and family data). It can, furthermore, manage longitudinal designs such as time series and growth models. For example, Dahly, Adair, and Bollen [22] developed a longitudinal latent variable medical model showing that maternal characteristics during pregnancy predicted children's blood pressure and weight approximately 20 years later while controlling for child's birth weight. Therefore, SEM can be used for a number of research designs.

A distinct advantage of SEM over conventional multiple regression analyses is that the former has greater statistical power (probability of rejecting a false null hypothesis) than does the latter. This is demonstrated in Budtz-Jørgensen's epidemiological study of benchmark calculations to exposure of environmental toxins [38]. They were able to show that SEM statistics were more sensitive to changes in toxin exposure than were regression statistics, which resulted in estimates of lower, or safer, exposure levels than did the regression analyses.

SEM has sometimes been referred to as causal modeling; however, caution must be taken when interpreting SEM results as such. Several conditions are deemed necessary, but not sufficient for causation to be determined. There must be an empirical association between the variables - they are significantly correlated. A common cause of the two variables has been ruled out, and the two variables have a theoretical connection. Also, one variable precedes the other, and if the preceding variable changes, the outcome variable also changes (and not vice versa). These requirements are unlikely to be satisfied; thus, causation cannot be definitively demonstrated. Rather, causal inferences are typically made from SEM results. Indeed, researchers argue that even when some of the conditions of causation are not fully met causal inference may still be justifiable [39].

### Weaknesses

As with any method, SEM has its limitations. Although a latent variable is a closer approximation of a construct than is a measured variable; it may not be a pure representation of the construct. Its variance may consist of, in addition to true variance of the measured variables, shared error between the measured variables. Also, the advantage of simultaneous examination of multiple variables may be offset by the requirement for larger sample sizes for additional variables to derive a solution to the calculations.

SEM cannot correct for weaknesses inherent in any type of study. Exploration of relationships among variables without a priori specification may result in statistical significance but have little theoretical significance. In addition, poor research planning, unreliable and invalid data, lack of theoretical guidance, and over interpretation of causal relationships can result in misleading conclusions.

## 4. Summary

With the development of SEM, medical researchers now have powerful analytic tools to examine complex causal models. It is superior over other correlational methods such as regression as multiple variables are analyzed simultaneously, and latent factors reduce measurement error. When used as an exploratory or confirmatory approach within good research design it yields information about the complex nature of disease and health behaviors. It does so by examining both direct and indirect, and unidirectional and bidirectional relationships between measured and latent variables. Despite the valuable contribution of SEM to research methodology, the researcher must be aware of several considerations to develop a legitimate model. These include using an appropriate research design, a necessary sample size, and adequate measures. Nevertheless, the theory and application of SEM and their relevance to understanding human phenomena are well established. In the context of medical research it promises the opportunity of examining multiple symptoms and health behaviors that, with model development and refinement, can be utilized to enhance our research capabilities in medicine and the health sciences.

## Competing interests

The authors declare that they have no competing interests.

## Authors' contributions

Both authors made substantial intellectual contributions to this paper. They have both read and approved the final manuscript.

## Authors' information

TNB is an Associate Professor in Medical Education and Research, Faculty of Medicine at the University of Calgary.

CV is a Professor in Medical Education and Research, Faculty of Medicine at the University of Calgary.

## Appendix

^a ^ANOVA and multiple regression analysis are instances of the General Linear Model.

^b ^*p = p (p+1)/2 can be used to determine the number of free parameters (*p) that can be estimated from the number of measured variables (p).

^c ^A model with equal values and parameters is said to be identified, and one with more values than parameters is overidentified; both models can be empirically assessed.

^d ^http://www.mvsoft.com/

http://www.ssicentral.com/lisrel/

http://www.statmodel.com/

http://www.spss.com/amos/

^e ^Diagnoses were based on the criteria listed for classification in both the third revised or fourth edition of the Diagnostic Statistical Manual (DSM-IV), which is an international classification system for mental health disorders in children and adults published by the American Psychiatric Association.
